# Participatory action research, mixed methods, and research teams: learning from philosophically juxtaposed methodologies for optimal research outcomes

**DOI:** 10.1186/s12874-018-0636-1

**Published:** 2018-12-12

**Authors:** Marguerite C. Sendall, Laura K. McCosker, Alison Brodie, Melissa Hill, Phil Crane

**Affiliations:** 1 School of Public Health and Social Work / Institute of Health and Biomedical Innovation, Queensland University of Technology (QUT), Victoria Park Road, Kelvin Grove, QLD 4159 Australia; 2 School of Social Science, University of the Sunshine Coast, Room D1.32, Locked Bag 4, Maroochydore DC, QLD 4558 Australia

**Keywords:** Quantitative, Qualitative, Mixed methods, Participatory action research (PAR), Research paradigms, Outdoor workers, Workplace health promotion, Sun protection

## Abstract

**Background:**

Workplace health interventions incorporating qualitative and quantitative components (mixed methods) within a Participatory Action Research approach can increase understanding of contextual issues ensuring realistic interventions which influence health behaviour. Mixed methods research teams, however, face a variety of challenges at the methodological and expertise levels when designing actions and interventions. Addressing these challenges can improve the team’s functionality and lead to higher quality health outcomes. In this paper we reflect on the data collection, implementation and data analysis phases of a mixed methods workplace health promotion project and discuss the challenges which arose within our multidisciplinary team.

**Methods:**

This project used mixed methods within a Participatory Action Research approach to address workers’ sun safety behaviours in 14 outdoor workplaces in Queensland, Australia, and elucidate why certain measures succeeded (or failed) at the worker and management level. The project integrated qualitative methods such as policy analysis and interviews, with a range of quantitative methods – including worker surveys, ultraviolet radiation (UVR) exposure measurement, and implementation cost analyses.

**Results:**

The research team found the integration of qualitative and quantitative analyses within the Participatory Action Research process to be challenging and a cause of tensions. This had a negative impact on the data analysis process and reporting of results, and the complexity of qualitative analysis was not truly understood by the quantitative team. Once all researchers recognised qualitative and quantitative data would be equally beneficial to the Participatory Action Research process, methodological bias was overcome to a degree to which the team could work cooperatively.

**Conclusions:**

Mixed methods within a Participatory Action Research approach may allow a research team to discuss, reflect and learn from each other, resulting in broadened perspectives beyond the scope of any single research methodology. However, cohesive and supportive teams take constant work and adjustment under this approach, as knowledge and understanding is gained and shared. It is important researchers are cognisant of, and learn from, potential tensions within research teams due to juxtaposed philosophies, methodologies and experiences, if the team is to function efficiently and positive outcomes are to be achieved.

## Background

In health outcomes research, it is important to use appropriate methods to obtain the best possible data with minimal bias [1]. Previous research to improve sun safety in the workplace has predominately used quantitative methods to study improvement in sun safety behaviours [2, 3], however, quantitative research alone does allow complex issues like sun safety behaviour to be fully understood. It offers little contextualised evidence explaining why certain sun safety interventions have or have not worked, and it cannot help identify essential elements to be included in multi-component sun safety health promotion interventions [2]. Mixed methods within a Participatory Action Research (PAR), as applied to workplace health promotion for sun safety, can provide a greater understanding which ensures realistic interventions to influence behavioural change in specific in settings [4]. However, the mixed methods approach can raise several challenges during planning, implementation and analysis stages [5–10].

In particular, teamwork has been identified as a key issue impacting on the potential effectiveness of mixed methods research [11]. Mixed methods teams can face a variety of challenges while designing actions and interventions at the methodological and expertise levels. Qualitative and quantitative researchers hold juxtaposed ontological or epistemological stances which make teamwork difficult or impossible [11, 12]. To overcome potential challenges, a successful mixed methods research team requires a good understanding of the nature of the research questions and the expertise required to address them [13]. Team members must be willing to learn each other’s approach to share knowledge, build trust, and develop a common language [13].

In this paper we reflect on the data collection, implementation and data analysis phases of a mixed methods workplace health promotion project addressing sun safety behaviours in outdoor workers. We discuss the challenges which arose within our multidisciplinary team in designing and implementing intervention components, and explain how we learnt to effectively minimise or overcome these challenges.

## Methods

### Project description

The aim of this project was to implement a comprehensive health promotion intervention using mixed methods within a Participatory Action Research (PAR) approach to influence the sun-related attitudes and behaviours of outdoor workers in workplace settings in Queensland, Australia. Recruitment and baseline characteristics are described in detail previously [14]. Briefly, 14 workplaces were recruited. These included small and large organisations across the rural, building and construction, public and local government sectors in geographically dispersed regions of Queensland. All the organisations employed outdoor workers, defined as individuals who work outdoors for most of the day on at least 5 days per week.

Each workplace nominated a workplace representative as a champion. The champions, with other workers, were invited to participate in the development of a plan for the suite of sun-safe health promotion interventions for the workplace. These plans were referred to as Sun Protection Action Plans and were developed over time in partnership with the research team. They encompassed six domains, as appropriate to, and based on data from, each workplace: policy, structure and environment, Personal Protective Equipment (PPE), education and awareness, role modelling, and skin examinations.

Using the principles and processes of a PAR approach, mixed methods were applied concurrently throughout the development of the Action Plans and data collection stages [9]. Two preliminary tools were used to engage workers and workplaces, identify the priority given to sun safety within each organisation, and gather a policy baseline. The first was a telephone-based screening tool, comprised of quantitative and qualitative questions about workplace demographics, locations and structures and existing workplace policies and procedures related to sun safety and UVR exposure. A second, more comprehensive situational analysis tool, conducted in person with the representative in each workplace, involved the systematic collection of detailed information about existing workplace sun safety policies, procedures and strategies,

The combination of data from these tools enabled a comprehensive picture of existing sun safety strategies and culture to be developed, to inform the sun safety interventions for each workplace. As the researchers were on-site to conduct the more comprehensive situational analysis, it was prudent to integrate additional research strategies targeting workers. For example, a discussion group with outdoor workers from each site was undertaken. The discussion group was guided by PAR principles to allow the discovery of information grounded in the workers’ realities. The discussion groups involved generating ideas from workers about strategies to promote and increase sun safety practices in their workplace. The discussion was transparent, free-flowing and allowed a ‘heads together’ way of thinking. It valued the workers’ inputs, took advantage of their existing skills, knowledge and resources and stimulated ideas. In most cases, supervisors were excluded from the discussion to allow workers to speak openly. All discussions were documented and transcribed to identify key themes.

Quantitative measures employed as the project developed included 1) the distribution of ultraviolet radiation (UVR) dosimeters to workers to measure their UVR exposure across one working day, and 2) a telephone survey with a sample of the workers from each workplace, to collect information about workers’ demographics, behaviours and attitudes related to sun exposure and protection in the workplace, knowledge of workplace sun protection policy, and perception of their workplaces’ level of support for sun protection. Similar data was collected during the research evaluation stage to allow for pre and post-intervention comparisons. Additional questions about workers’ perception of changes to workplace sun safety protocols were asked.

### Working with a diverse team

This project was conducted by a multidisciplinary team of quantitative and qualitative researchers with various expertise in epidemiology, public health, health promotion, health economics, and the social sciences.

The team consisted of eight researches and two professional staff. All researchers held a doctoral degree. Five researchers were quantitative experts, including the Project Lead. The three qualitative methodologists were assigned an equal number of geographically feasible and like workplaces to undertake fieldwork with a Research Assistant. The Project Office consisted of two contracted professional staff – a Project Manager and a Research Assistant. Casual professional support staff was provided when needed, for example, undertaking surveys. Six researchers were located within travelling distance of the project office. One researcher was located in another Australian city and one researcher was located in Canada. The team met once a fortnight in the planning phase, and once a month during fieldwork. Six team members attended in person and two team members attended by Skype. Most team members attended most meetings. Two team members attended meetings irregularly. Each meeting was guided by an agenda developed by the project lead.

Before the project commenced, the research team set the foundation for how they would use mixed methods within the PAR approach to address the research question. The research team needed to collectively define the meaning of mixed methods and PAR to create a common language. This was complicated by the diversity of methodological expertise and experience with PAR as a research approach. Through early discussions, the researchers learnt from each other to achieve an understanding and consensus about what data each method was collecting within the PAR process, and how this information would be useful to all relevant parties: the funding body, the health promotion community, the workers and the workplaces and the skin cancer research community. This was a healthy debate which required researchers from both methodologies to consider the scope and sequence of the research. Once all researchers recognised qualitative and quantitative data would be equally beneficial to the PAR process, methodological bias was overcome to a degree to which the team could work cooperatively.

A methods paper for use by the research team was then drafted by the project manager to outline the selected mixed methods, explain how these would be integrated, and indicate the logical sequence of PAR activities. This paper allowed the research team to conceptualise the mixed methods not as designs, but as a set of interactive parts [15]. This methods paper was crucial in guiding the research team in applying the principles and processes of PAR in the project.

## Results

Researchers were encouraged to contribute to conversations from their own methodological perspective, and make these perspectives available for discussion and debate. These shared learnings fostered respect amongst the team and enabled effective information sharing because researchers felt safe voicing concerns [16]. As respect from learning grew among the team, leadership became more collaborative and the hierarchy underpinning the team dissolved. A separate language was not created but the focus remained on understanding and learning the language used by other researchers to keep the underlying methodologies of qualitative and quantitative research clear. This process of learning was a fundamental step in facilitating the team’s effective use of the mixed methods within the PAR approach.

There was, however, tension within the research team about how best to integrate qualitative and quantitative data collection methods without exceeding the project timeline. Such tension regarding data methods integration is common in mixed methods research and is an often-cited barrier to conducting research of this type [5, 9]. After listening, reflecting and learning it was agreed data collection had to be resilient and flexible [16], combining qualitative and quantitative methods to ensure each was not wholly dependent on the other. For example, as the team became aware of rising tensions, ‘methodology’ was tabled as an agenda item at each team meeting. This ensured there was explicit permission and opportunity for team members to ask questions and clarify concerns about underlying methodological reasoning, engage in discussion about integrating approaches and ensuring rigour. This helped avoid the added complexity of problems and potential setbacks normally associated with the interdependency of multiple methods [16] throughout the PAR process.

The team’s different approaches to mixed methods had a negative impact on the data analysis process and reporting of results. The complexity of qualitative analysis was not truly understood and recognised by the quantitative team. For example, analysis of quantitative results was prioritised to meet the final report deadline. The analysis of qualitative findings was left until the quantitative results were completed. The limited time for qualitative analysis impacted a rigorous analytical process and the opportunity to present quantitative and qualitative outcomes as a comprehensive integrated whole. Subsequently, the project’s qualitative and quantitative outcomes were reported separately in the final report [17, 18].

A key philosophy of this project was the participatory and collaborative nature of planning between workers and each workplace and the research team. The qualitative methodologist worked directly with workplaces acting as a link to the project office. Over the 12-month intervention period, these team members developed rapport with the workplaces arriving at a deep understanding of the workplace context. This is consistent with the PAR approach. Time was taken to consider outcomes from the situational analysis tools and discussion groups, to negotiate meaning with stakeholders, to build a shared understanding of the pre-intervention data and to decide upon the most effective strategies for the workplace. This involved the research team sharing and learning from insights of existing practices and piecing together a plan which best suited individual workplaces. Throughout the intervention period, the research team met regularly to discuss fieldwork progress. This process was challenged however, because 1) some team members did not attend meetings, 2) team meetings were dominated by quantitative experts and 3) the same challenges were discussed from one meeting to the next.

The Sun Protection Action Plans were implemented and progress monitored by the research team, with all nuances, key outcomes, barriers and facilitating factors documented in a case study design. This created further discussion and learning amongst the research team. Team members were required to ‘step back’ from their own area of expertise and genuinely endeavour to understand another’s view and to situate that view within the projects. This was often difficult because not all team members had the experience of working in mixed methods teams. The team faced the challenges of being true to the PAR process by not confining the development of case studies to rigid boundaries, yet ensuring the case studies reflected consistent themes. For example, a proposed research design involved grouping workplaces to control and case. Case workplaces would be assigned a suite of interventions. This design is not viable due to the highly contextual nature of workplaces but remained on the team meetings’ agenda despite discussions. The case studies aimed to consider the voice and perspective of management (policy analysis, the relevant groups of workers (survey research) and the interaction between them. Case study development was a joint exercise between the workers and each workplace and the research team and no unresolved conflicts arose during their construction.

## Discussion

There is a lack of research about optimal ways for teams to function in mixed method studies [19]. A significant learning from this project which could benefit the functionality and cohesiveness of mixed methods research teams is an understanding of the importance of commencing from a platform of social inquiry and extending to a common analytical space, rather than relying on individual investigators’ philosophical assumptions. For example, there was no early discussion about the weighting of methodologies – was this a quantitative project, with some qualitative injury, or was this a qualitative project, with some quantitative inquiry? Team members may have assumed equal or unequal weighting. Either way, the integration of these analyses within the PAR process was challenging. For example, there was an unspoken but obvious positioning between the methodologies. Qualitative methodology was highly positioned within each workplace and fieldwork and quantitative methodology was highly positioned in the project office and the project our-outcomes. The analysis strategy originally involved use of qualitative data to interpret the quantitative results and then the integration and comparison of qualitative and quantitative phases at the beginning and the end of the project. As the project developed, the reporting of quantitative results was prioritised, particularly at the evaluation stage.

### Lessons learnt and future challenges

Ineffective communication underpinned by a lack of a shared language or ‘methodological disrespect’ may impede successful teamwork [16]. This issue of compatibility between research philosophies arose while the project was being designed, and required the research team to listen, reflect and learn about each other’s perspectives. Although consensus was reached about the integration of quantitative and qualitative methodologies within a PAR approach, there was general learning amongst the researchers that a shift away from a preoccupation with explicit assumptive differences among paradigms, and toward other characteristics of social inquiry traditions was required. Paradigms are not universally accepted as necessary among mixed methods researchers as they can marginalise other beliefs or force researchers to adhere rigidly to a set of beliefs [20]. However, they are useful to guide inquiry, especially for novice mixed methods researchers or teams. Where paradigms are applied, they are regarded as not static, concrete entities which restrict a research process [21] but rather, a system of beliefs and practices which influence what questions are asked [21]. Moving away from qualitative or quantitative methodologies and towards those inherent in mixed methods within a PAR approach allows researchers to transition beyond the methodological binary and ‘paradigm wars’ that have characterised social science research for several decades [20].

This project subscribed most fully, though not in a contrived way, to the transformative-emancipatory paradigm as outlined by [22]. This perspective is characterised by the intentional collaboration with minority and marginalised groups to address a research problem [22]. While it was not the intention of the research team to subscribe to a paradigm, the use of action research to guide this project meant this happened incidentally. Purposeful use of a guiding paradigm may have allowed the research team to learn from one another and reach a consensus about the use of qualitative and quantitative methods earlier in the planning stages of the project.

## Conclusion

The use of mixed methods within a PAR approach will continue to expand across disciplines and fields. It is important researchers are cognisant of, and learn from, potential tensions within research teams due to juxtaposed philosophies, methodologies and experiences. If positive outcomes are to be achieved, learning from each other for the efficient functioning of the research team is just as important as the effective integration of qualitative and quantitative research methods. Cohesive and supportive teams take constant work and adjustment as knowledge and understanding is gained and shared. Discussion of strategies to facilitate team cohesiveness in mixed methods projects is still widely unavailable in the literature.

Even though challenges were faced by our research team at every phase of the project, mixed methods within a PAR approach allowed the team to discuss, reflect and learn from each other, resulting in broadened perspectives beyond the scope of any single research methodology.

## Abbreviations

PAR: Participatory action research
PPE: Personal protective equipment
UVR: Ultraviolet radiation
